# Resistance Training for Diabetes Prevention and Therapy: Experimental Findings and Molecular Mechanisms

**DOI:** 10.1155/2013/805217

**Published:** 2013-12-22

**Authors:** Barbara Strasser, Dominik Pesta

**Affiliations:** ^1^Institute for Nutritional Sciences and Physiology, University for Health Sciences, Medical Informatics and Technology, A-6060 Hall in Tirol, Eduard Wallnoefer-Zentrum 1, Austria; ^2^Department of Internal Medicine, Yale University School of Medicine, 333 Cedar Street, New Haven, CT 06510, USA

## Abstract

Type 2 diabetes mellitus (T2D) is characterized by insulin resistance, impaired glycogen synthesis, lipid accumulation, and impaired mitochondrial function. Exercise training has received increasing recognition as a cornerstone in the prevention and treatment of T2D. Emerging research suggests that resistance training (RT) has the power to combat metabolic dysfunction in patients with T2D and seems to be an effective measure to improve overall metabolic health and reduce metabolic risk factors in diabetic patients. However, there is limited mechanistic insight into how these adaptations occur. This review provides an overview of the intervention data on the impact of RT on glucose metabolism. In addition, the molecular mechanisms that lead to adaptation in skeletal muscle in response to RT and that are associated with possible beneficial metabolic responses are discussed. Some of the beneficial adaptations exerted by RT include increased GLUT4 translocation in skeletal muscle, increased insulin sensitivity and hence restored metabolic flexibility. Increased energy expenditure and excess postexercise oxygen consumption in response to RT may be other beneficial effects. RT is increasingly establishing itself as an effective measure to improve overall metabolic health and reduce metabolic risk factors in diabetic patients.

## 1. Introduction 

The global epidemic of obesity has contributed to a concomitant increase in the prevalence of type 2 diabetes mellitus (T2D). The number of people with T2D increased from 153 million in 1980 to 347 million in 2008 and is projected to reach 550 million worldwide by the year 2030 [1]; therefore, the disease is a growing public health concern and major socioeconomic burden [2]. Type 2 diabetes mellitus and prediabetic conditions such as impaired glucose tolerance are characterized by varying levels of insulin resistance causing hyperglycemia. Disturbances in glucose and insulin metabolism may not be a normal characteristic of aging but are rather associated with obesity and physical inactivity [3]. Exercise training has received increasing recognition as a cornerstone in the prevention and treatment of T2D. Based on the current position statement from Exercise and Sport Science Australia, it is recommended that patients with T2D or prediabetes accumulate a minimum of 210 min per week of moderate-intensity exercise or 125 min per week of vigorous intensity exercise with two or more resistance training (RT) sessions per week included into the total time [4]. It is well established that endurance exercise brings about numerous beneficial physiological changes such as increased maximal oxygen utilization (VO_2max_) and cardiopulmonary fitness, as well as peripheral adaptations such as increased fatty acid transport and oxidation, improved capillary density, and mitochondrial capacity [5]. Positive health effects of RT, however, are only relatively recently being recognized and characterized. Before the year 1990, neither the American Heart Association nor the American College of Sports Medicine (ACSM) included guidelines for recommendation of RT for exercise training and rehabilitation. It was in 1990 when the ACSM recognized RT as a contributing factor to a comprehensive fitness program for healthy adults of all ages. The current position statement for exercise and type 2 diabetes by the ACSM and the American Diabetes Association (ADA) recognizes the beneficial effects of RT and recommends RT at least twice a week in addition to aerobic training for persons with T2D [6]. Evidence from randomized controlled trials has shown that RT improves glycemic control in patients with T2D, increases glucose disposal, and even improves the lipid and cardiovascular disease risk profile of patients with T2D [7, 8]. Furthermore, the Health Professionals Follow-up Study examined the association of RT in the primary prevention of T2D and found a 34% lower risk of T2D in men, independent of aerobic exercise [9]. However, there is limited mechanistic insight into how these adaptations occur. The purpose of this review is to (1) provide an overview of the intervention data on the impact of RT on glucose metabolism, and (2) to hypothesize about the potential role of RT to combat metabolic dysfunction in patients with T2D by describing possible cellular and molecular mechanisms. We included both well-designed randomized and nonrandomized controlled RT trials, but only those non-randomized controlled RT trials were included that were frequently cited by others or had other indicators of good internal controls.

## 2. Aging, Obesity, and Insulin Resistance 

Biological ageing is typically associated with a progressive increase in body fat mass and a loss of lean body mass, particularly skeletal muscle termed sarcopenia [10]. Skeletal muscle mass—the primary site of glucose and triglyceride disposal—declines at a rate of 3 to 8% each decade after the age of 30 [11] which may lead to a rise of risk developing glucose intolerance and T2D [12]. Due to the metabolic consequences of diminished muscle mass with aging, including lowered resting metabolic rate, reduced glucose uptake, and capacity for lipid oxidation, it is understood that normal aging and/or decreased physical activity may lead to a higher prevalence of metabolic disorders. Evidence from epidemiological studies has shown that muscular strength is inversely related to both the metabolic syndrome and all-cause mortality [13, 14]. Visceral fat increases by over 300% between the ages of 25 and 65 years, and this creates an increased risk for the development of T2D and cardiovascular disease (CVD) in adults with normal body mass index [15]. The distribution of excess fat in the abdominal region modifies the health risk profile. In contrast, excess adiposity in the periphery does not appear to increase the risk of developing CVD [16]. Intra-abdominal fat compared to total body fat correlates significantly better with triglycerides, systolic and diastolic blood pressure and is expected to decrease the sensitivity of target tissues to insulin [17, 18]. Intra-abdominal obesity is an important risk factor for low-grade inflammation. The adipokines adiponectin and tumor necrosis factor alpha (TNF-*α*) play a role in body fat distribution and correlate with aging and insulin resistance [19]. Serum adiponectin levels are negatively associated with fat mass and reduced adiponectin levels play a causal role in the development of insulin resistance. Adiponectin is an insulin-sensitizing protein and hypoadiponectinemia is therefore associated with obesity, insulin resistance, and type 2 diabetes [20]. Adiponectin is believed to activate 5′-AMP-activated protein kinase (AMPK), which activates insulin-independent glucose uptake by the muscle [21]. TNF-*α* is an inflammatory cytokine secreted by adipose tissue, with high concentrations of TNF-*α* being linked to obesity and insulin resistance [22].

In obesity and T2D fatty acid metabolism in skeletal muscle is dysregulated, resulting in the accumulation of lipids within the muscle cell [23]. These intramuscular lipid products interfere with insulin signaling within the muscle cell thereby contributing to insulin resistance [24]. Even a single bout of RT increases VLDL-TG plasma clearance rate by 26% as compared with the rest thereby reducing the residence time of VLDL-TG in the circulation of untrained men [25]. This effect may be mediated by increased VLDL-TG catabolism and hydrolysis during recovery by augmentation of lipoprotein lipase (LPL) gene expression and activity in the skeletal muscle [26]. In aging and insulin-resistant conditions the ability of insulin to stimulate GLUT4 translocation decreases, resulting in a reduced GLUT4 content at the plasma membrane [27]. Exercise-induced, contraction-mediated GLUT4 translocation to the muscle membrane is independent of insulin and occurs through calcium/calmodulin-dependent protein kinase IV and, secondarily, through AMPK, which induces expression of PGC-1*α*, a transcriptional coactivator that is essential for mitochondrial biogenesis [28, 29]. A 40% reduction in mitochondrial function with aging, obesity, and T2D may contribute to declines in glucose uptake and the development of insulin resistance, possibly arising from increases in intracellular lipid stores [30]. Once taken up by the cell, glucose can either be oxidized to carbon dioxide and water or converted to glycogen, the latter being regulated by glycogen synthase. Patients with T2D show significantly lower glycogen synthesis rates compared to healthy controls [31], although obese-diabetic patients tended to have higher muscle glycogen content [32].

**Figure 1:**
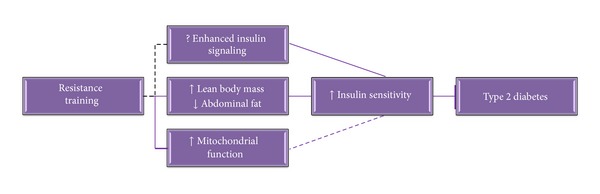
Schematic diagram of future directions to determine mechanisms by which resistance training (RT) increases insulin sensitivity and prevents type 2 diabetes (T2D).

Taken together, evidence indicates metabolic dysfunction in skeletal muscle in patients with T2D, characterized by insulin resistance, impaired glycogen synthesis, lipid accumulation, and impaired mitochondrial function. Aging *per se* has an influence on skeletal muscle loss, but the metabolic impairment and functional losses can be largely counteracted by exercise training, especially by RT. Emerging research suggests that RT may influence age-related physiological changes and may impose potent and unique benefits in T2D. An overview of how RT may impact diabetes risk is presented graphically in Figure 1.

## 3. Implications of Resistance Training 

Recent evidence indicates that RT has the power to combat metabolic dysfunction in obese, T2D patients [8, 33–35]. However, only one prospective study has examined the association of RT with risk of T2D. According to the Health Professionals Follow-up Study who observed >32,000 men over a period of 18 years, subjects engaging in RT over >150 min/week showed a 34% reduction in risk of T2D, after adjustment for aerobic activities and body mass index (BMI) [9]. In addition, those with a BMI ≥ 30 who engaged in RT ≥ 150 minutes per week exhibited an estimated 60% reduction in risk [9]. Thus, part of the beneficial effect of RT was mediated by abdominal adiposity as demonstrated in a previous analysis from the Health Professionals Follow-up Study [36]. Another interesting observation was the 40% reduction is risk in those without a family history of T2D; however, no effect was noted in those with family history. Resistance training may assist prevention and management of T2D by decreasing visceral fat and inflammatory markers [37, 38], increasing the density of GLUT4 [39], and improving insulin sensitivity as noted in a wide range of study groups and discussed in two recent reviews by Hurley et al. [34] and Flack et al. [40]. Acutely performed multiple sets and even single sets of RT increased 24-hour postexercise insulin sensitivity in subjects with impaired fasting glucose [41]. Three months of RT in obese, adolescent boys resulted in a significant reduction of total and visceral fat and intrahepatic lipid as well as increased insulin sensitivity compared with controls [42]. Although a recent systematic review reveals only a slight decrease in visceral fat with RT as the sole intervention in healthy or overweight/obese adults [35], studies have shown that regular RT is effective in reducing abdominal fat among individuals with T2D, even without significant weight loss [43, 44]. Thus, RT is contributing to the decrease of one of the major risk factors for insulin resistance. Resistance training can improve glycemic control and insulin sensitivity [45], likely even more than aerobic endurance training [46, 47]. In subjects with T2D, improvements in insulin sensitivity with RT, compared to sedentary control, have been noted without a change in maximal oxygen uptake (VO_2max⁡_), weight loss, or body composition [48]. It is possible that an increase in lean body mass after RT may be an important mediator of the improved glycemic control. An increase in the number of GLUT4 transporters is discussed specifically, because the transporter protein GLUT4 expression at the plasma membrane is related to fibre volume in human skeletal muscle fibres [49]. However, increased muscle mass was not associated with improvement in glycemic control in one of our in-house studies [50]. One possible reason is that improvement in glycemic control is not only dependent on muscle mass change but also on the consequence of intrinsic alterations in the muscle. Holten et al. reported improved insulin action by increased protein content of GLUT4, insulin receptor, protein kinase B-*α*/*β*, and glycogen synthase after six weeks of one-leg RT while the untrained leg remained unchanged [39]. Augmented glycemic control thus reduces the amount of insulin necessary to accomplish the clearance of a given amount of glucose. Castaneda et al. found that 16 weeks of RT three times per week increased muscle glycogen by 32% in older adults with T2D, whereas the control group experienced a significant reduction in muscle glycogen [51]. Resistance training can improve glucose transport in both normal and insulin-resistant skeletal muscle by enhancing the activation of the insulin signaling cascade [52]. These training-induced alterations improve the metabolic profile of the skeletal muscle and can occur independently of significant increases in skeletal muscle mass [53]. Dunstan et al. found decreased baseline glucose and insulin values in diabetic patients after 8 weeks of circuit weight training, 3 times a week at 55% 1RM when compared to nonexercising controls [54]. It seems that multiple RT programs are capable of improving insulin sensitivity. However, the effect of RT on insulin action is lost when the training is stopped [55]. Furthermore, because of reduced adherence and training intensity, home-based RT is less effective for maintaining glycemic control than supervised RT [56].

Glycosylated hemoglobin (HbA1c) is the most accepted parameter for assessing long-term glycemic control and is strongly associated with risk of diabetes, CVD, and death [57]. In a recent meta-analysis, aerobic, resistance, and combined exercise training were found to be associated with HbA1c reductions of 0.67% following 12 or more weeks of training [7]. In another meta-analysis of 10 included supervised resistance exercise studies, RT reduced HbA1c by 0.48% [8]. Bacchi et al. recently showed that 4 months of resistance and aerobic training were equally effective in improving hepatic fat content, insulin sensitivity, body fat mass, and HbA1C in adults with type 2 diabetes and NAFLD [58]. Ideally, both aerobic endurance training and RT should be combined in the exercise prescription for T2D and prediabetes. Recent research has identified that combining both forms of exercise of an equal caloric expenditure (12 kcal/kg/week) among combined and separate AET and RT groups may lead to greater glycemic control benefits (−0.34%) that were not found with either type of training alone [59]. It is recommended that two or more RT sessions per week (2–4 sets of 8–10 repetitions) should be included in the total 210 or 125 min of moderate or vigorous exercise, respectively [4].

Mitochondrial dysfunction and fat accumulation in skeletal muscle (increased intramyocellular lipid content) have been linked to development of T2D [60, 61]. Only very few interventions evaluated the effects of a RT program on muscle lipid content in patients with T2D. Praet et al. reported in individuals with T2D no change in intramyocellular lipid content and muscle oxidative capacity after 10 weeks of RT, three times a week at 60% 1RM, combined with interval training (4–8 cycling bouts of 30 s, at 50–60% of the maximum achieved workload alternated with 60 s of unloaded cycling) [62]. In another study, 44 women with T2D were randomly assigned to three groups for a period of 12 weeks: RT (using elastic bands) at 40–55% of maximal strength five times per week; aerobic endurance training (walking) at moderate intensity five times per week; and control (asked to maintain a sedentary lifestyle) [63]. Changes in subcutaneous, subfascial, and intramuscular adipose tissue were similar among the three groups, but retinol-binding protein-4 which is linked to adipose tissue and insulin sensitivity in diabetics decreased significantly only with RT [63]. Resistance exercise training is an important countermeasure for aging-associated muscle weakness and increases muscle strength and function in older adults, in association with a reduction in markers of oxidative stress and an improvement in mitochondrial function [64]. Pesta and colleagues demonstrated that 10 weeks of RT enhanced mitochondrial respiration to the same extent as aerobic training in skeletal muscle of lean, previously sedentary adults [65]. The objective of a very recent study was to investigate effects of different types of exercise on mitochondrial content and substrate oxidation in individuals with T2D [66]. Patients were randomized to RT, aerobic training, combined training, or nonexercise control. The first significant finding of this investigation was the clear demonstration of an RT-induced increase in mitochondrial content in the skeletal muscle of T2D patients after 9 months of training, and these changes were significantly associated with clinical improvements (i.e., HBA1c, VO_2max_). Furthermore, combined training improved all measures of lipid and carbohydrate oxidation as well as mitochondrial content and enzyme activity [66]. Thus, available evidence strongly suggests that the lower mitochondrial capacity associated with obesity, T2D, and aging is not an irreversible lesion. Either aerobic or resistance exercise, but not weight loss, has the power to improve mitochondrial content and possibly the function of the individual mitochondrion [67]. However, the link between mitochondrial oxidative capacity and insulin resistance remains inconclusive and requires further research.

Resistance exercise further increases excess postexercise oxygen consumption (EPOC) [68]. This increase in VO_2_ after a RT session increases energy expenditure during the recovery period. EPOC after exercise is related to utilization of fat as fuel which is beneficial for weight loss [69]. Resistance training itself and EPOC, which, according to some studies, [70, 71] is higher after RT than after aerobic training, both contribute to an increase in energy expenditure and hence constitute important systemic factors which add to metabolic health.

## 4. Cellular and Molecular Mechanisms 

It is well established that endurance exercise brings about numerous beneficial physiological changes such as increased maximal oxygen uptake (VO_2max_) and cardiopulmonary fitness, as well as peripheral adaptations such as increased fatty acid transport and oxidation, improved capillary density, and mitochondrial capacity [72]. In this section, the molecular mechanisms that lead to adaptation in skeletal muscle in response to RT and that are associated with possible beneficial metabolic responses will be discussed.

One hallmark of strength and resistance training is an increase in muscle strength and muscle mass, mediated mainly via muscle hypertrophy and neuromuscular remodeling. As already mentioned above, RT can increase insulin sensitivity by qualitative changes independent of a gain in muscle mass. It is well established, however, that body sensitivity to insulin is directly proportional to muscle mass. Gain in muscle mass must therefore remain an important goal in RT for patients. In addition, increase of lean mass leads to an increased resting metabolic rate [73], therefore possibly triggering an upward spiral of metabolic health.

### 4.1. Mechanisms Responsible for an Increase in Muscle Mass

One of the main pathways responsible for muscle hypertrophy via increased protein synthesis is the IGF-1/PI3K/AKT pathway. Its ligand, insulin-like growth factor 1 (IGF-1), is a well characterized growth promoting factor and plays a crucial role in muscle growth and regeneration. Upon ligand binding to the IGF-1 receptor, several intracellular signaling events finally lead to a phosphorylation of Thr308 residues on AKT to activate AKT [74]. Activated AKT can then act on downstream proteins such as the forkhead box O (FoxO) transcription factor family. FoxO is an important player involved in integrated cellular responses such as protein metabolism [75]. AKT mediates FoxO phosphorylation and repression of this transcription factor which leads to inhibition of protein degradation.

AKT stimulates protein synthesis via mammalian target of rapamycin (mTOR). The mTOR complex consists of two multiprotein complexes, mTOR complex 1 (mTORC1) and mTOR complex 2 (mTORC2), each of them exhibiting different sensitivities to rapamycin [76]. Activation of mTORC1 leads to phosphorylation of ribosomal protein S6 as well as other factors involved in translation initiation and elongation which results in increased protein synthesis [74].

Next to a change in cross-sectional area and muscle mass, RT also induces a shift in the muscle fiber type distribution from low-oxidative type 2x muscle fibers to moderate-oxidative, more insulin-sensitive type 2a muscle fibers [77].

### 4.2. Mechanisms Mediating Increased Glucose Clearance

Important for the beneficial metabolic effect of RT is stimulation of glycogen synthesis via inhibition of glycogen synthase kinase 3*β* (GSK3) by AKT [78]. GSK3*β* regulates glucose storage in the form of glycogen. AKT can inhibit GSK3 by phosphorylating it at a serine residue (Ser9 in GSK3*β*). Inhibition of GSK3 promotes activation of glycogen synthase (GS) which contributes to the stimulation of glycogen synthesis. Glycogen synthase is the enzyme responsible for catalyzing the *α*(1→4) linkage in the formation of glycogen and is therefore important for nonoxidative glucose disposal. Increased AKT-mediated GS activity is therefore an important adaptation towards glycemic control in response to RT.

It has been observed that AMPK activity is increased as an acute exercise phenotype-specific response to RT [79]. This activation leads to reduced phosphorylation of 4E-BP1 and decreased mTOR signaling. The AMPK-mediated inhibition of mTOR may prevent muscle protein synthesis during resistance exercise. After exercise, inhibition is released and protein synthesis can occur in the muscle [79]. This transient activation of AMPK could possibly lead to phosphorylation of target proteins involved in a number of metabolic pathways which result in an increase of ATP generating pathways such as glucose uptake via increased GLUT4 translocation and fatty-acid oxidation [80]. Hyperinsulinemia decreases beta-oxidation in insulin resistant subjects and therefore reduces the utilization of fatty acids [81]. The insulin-sensitizing effect of RT releases the brake on beta-oxidation and contributes to improved metabolic flexibility and a more balanced utilization of fatty acids as substrates. Increased insulin sensitivity might therefore contribute to increased lipid clearance from the blood. Increased insulin receptor protein expression in response to RT might be another important adaptation responsible for the insulin-sensitizing effect of training [39]. These adaptations might be responsible for restoring metabolic flexibility in T2D in response to RT.

## 5. Conclusion

There is good evidence that RT improves insulin sensitivity and glucose tolerance and therefore seems to be an effective measure to improve overall metabolic health and reduce metabolic risk factors in diabetic patients. Detailed mechanisms of RT induced adaptations that contribute to an improved metabolic profile remain elusive. It also remains to be determined whether the mechanisms by which RT improves glycemic control are the same as those that affect improvements after endurance training. Increased energy expenditure and excess postexercise oxygen consumption in response to RT may be important systemic factors contributing to metabolic health. The beneficial effects of RT are promising. This is of significant interest as RT can be viewed as a suitable training modality in our time-poor society. In contrast to traditional high-volume endurance training, high-intensity/low-volume RT can be a time-efficient strategy to offer metabolic benefits. Positive effects of RT not only benefit diabetic patients but can also significantly improve quality of life of the elderly who are often suffering from sarcopenia and muscle weakness.
